# Persistent circulation of genotype D coxsackievirus A2 in mainland of China since 2008

**DOI:** 10.1371/journal.pone.0204359

**Published:** 2018-09-20

**Authors:** Qian Yang, Xinrui Gu, Yong Zhang, Haiyan Wei, Qi Li, Huan Fan, Yi Xu, Jie Li, Zhaolin Tan, Yang Song, Dongmei Yan, Tianjiao Ji, Shuangli Zhu, Wenbo Xu

**Affiliations:** 1 WHO WPRO Regional Polio Reference Laboratory and National Health Commission Key Laboratory for Medical Virology, National Institute for Viral Disease Control and Prevention, Chinese Center for Disease Control and Prevention, Beijing, People's Republic of China; 2 RCSC National Training Center, Beijing, People's Republic of China; 3 Henan Center for Disease Control and Prevention, Zhengzhou, People's Republic of China; 4 Hebei Center for Disease Control and Prevention, Shijiazhuang, People's Republic of China; 5 Jiangsu Center for Disease Control and Prevention, Nanjing, People's Republic of China; 6 Shaanxi Center for Disease Control and Prevention, Xi'an, People's Republic of China; 7 Beijing Center for Disease Control and Prevention, Beijing, People's Republic of China; 8 Tianjin Center for Disease Control and Prevention, Tianjin, People's Republic of China; 9 Medical School, Anhui University of Science and Technology, Huainan, People’s Republic of China; University of Hong Kong, HONG KONG

## Abstract

Coxsackievirus A2 (CV-A2) has emerged as an important etiological agent in the hand, foot, and mouth disease and herpangina pathogen spectrum because of its high global prevalence. In the present study, we investigated the evolutionary dynamics of CV-A2 circulating in China. We analyzed a total of 163 entire *VP1* sequences of CV-A2, including 74 sequences generated from the present study and 89 sequences collected from the GenBank database. Phylogenetic analysis based on the entire *VP1* nucleotide sequences confirmed the persistent circulation of the predominant genotype D in mainland of China since 2008. Cluster analysis grouped the sequences into two distinct clusters, clusters 1 and 2, with most grouped under cluster 2. After 2012, cluster 1 was gradually replaced by cluster 2. Results of Bayesian Markov chain Monte Carlo analysis suggested that multiple lineages of genotype D were transmitted in mainland of China at an estimated evolutionary rate of 6.32×10^−3^ substitutions per site per year, which is consistent with the global evolutionary rate of CV-A2 (5.82×10^−3^ substitutions per site per year). Continuous transmission and evolution of CV-A2 resulted in the genetic polymorphism.

## Introduction

Enteroviruses (EVs) are important etiological agents that can cause a wide spectrum of diseases in young children, ranging from febrile illness, hand, foot, and mouth disease (HFMD), herpangina, meningitis to encephalitis, and even death [1]. EVs belong to the genus *Enterovirus* in the family *Picornaviridae* and order *Picornavirales*. Human EVs consist of four species, namely, EV-A, EV-B, EV-C, and EV-D. The EV genome encodes 11 proteins, including VP1 to VP4, 2A to 2C, and 3A to 3D. The *VP1* coding region is responsible for inducing the human neutralizing antibody response and has been found to correlate well with identified serotypes [2–4]. Coxsackievirus A2 (CV-A2), which belongs to species EV-A, has emerged as an active pathogen that has been implicated in several HFMD and herpangina outbreaks worldwide since 2008, including in Taiwan in 2008 [5], Hong Kong in 2012 [6], Thailand in 2015 [7], and Hangzhou, mainland of China in 2015 [8]. In this study, we analyzed entire *VP1* nucleotide sequences collected from 2008 to 2017 to investigate the evolutionary dynamics of emerging CV-A2 in mainland of China. We used data collected from the national virologic surveillance programs to study the evolutionary genetics of CV-A2 genotypes in China within a 10-year time span.

## Materials and methods

### Ethics statement

The present study did not involve human participants or human experiments. The only human-derived materials used were stool samples, throat swab samples, and rectal swab samples collected from HFMD patients for public health purposes under the programs of the Ministry of Health, P. R. of China. Written informed consents for the use of clinical samples were obtained from the parents of the children enrolled in the study. This study was approved by the second session of the Ethics Review Committee of the National Institute for Viral Disease Control and Prevention (IVDC), Chinese Center for Disease Control and Prevention. All experimental protocols were approved by the IVDC, and the procedures were carried out in accordance with the approved guidelines.

### Virus isolation

Clinical specimens (stool samples, throat swab samples, and rectal swab samples) were collected from patients with HFMD in 13 provinces (municipalities) throughout mainland of China (Jilin, Gansu, Guizhou, Hebei, Hunan, Jiangxi, Shaanxi, Shandong, Jiangsu, Hainan, and Qinghai provinces and Beijing and Tianjin municipalities). All samples were processed according to standard and previously described protocols [9]. Real-time reverse transcription-polymerase chain reaction (RT-PCR) was performed to screening for EV-A71, CV-A16, and other EVs as previously described [10]. 3001 non-EV-A71 and non-CV-A16 EV positive samples were selected. Viruses were isolated from original clinical specimens by propagation in human rhabdomyosarcoma (RD) and human larynx carcinoma (HEp-2) cells following conventional methods [11]. These two cell lines were obtained from the WHO Global Poliovirus Specialized Laboratory, USA and were originally purchased from the American Type Culture Collection. The CV-A2 strains used in this study were isolated between 2012 and 2017.

### *VP1* sequencing of CV-A2 isolates

Viral RNA was extracted from non-EV-A71 and non-CV-A16 EV-positive samples using a QIAamp Viral RNA Mini Kit (Qiagen, Valencia, CA, USA). Reverse transcription polymerase chain reaction (RT-PCR) was performed to amplify the entire *VP1* capsid region (885 nucleotides) using PrimeScript One Step RT-PCR Kit Ver. 2 (TaKaRa, Dalian, China) and previously designed primers (forward (486 or CV-A2-2787-S) and reverse (488 or CV-A2-3618-A) primers) [12]. Preparation of RT-PCR reactions and amplification profiles were based on previously reported protocols. PCR products were purified using the QIAquick PCR Purification Kit (Qiagen, Germany). The amplicons were bi-directionally sequenced on an ABI 3130 Genetic Analyzer (Applied Biosystems, USA) [13].Sequences were deposited in GenBank under the accession numbers MG926783–MG926810.

### Phylogenetic analysis of CV-A2 sequences

In combination with the 74 entire CV-A2 *VP1* nucleotide sequences determined in this study, 89 entire CV-A2 *VP1* sequences from the GenBank database were collected prior to December 31, 2017. Thus, the current dataset comprised a total of 163 strains (data in S1 Table). Alignment of the nucleotide sequences of the CV-A2 strains was performed using BioEdit sequence alignment editor software (version 5.0). Maximum likelihood (ML) trees were estimated using the best-fit Kimura 2-parameter model of nucleotide substitution in Mega software (version 5.03) [14]. Branch lengths of the dendrogram were determined based on the tree topology and the majority rule consensus of 1,000 bootstrap replicates. Bootstrap values greater than 80% were considered statistically significant for grouping.

### Evolutionary analysis of CV-A2

The temporal structure of the data was tested with TempEst [15].To avoid oversampling, duplicate sequences from viruses collected at the same time point and location were discarded. The nucleotide substitution model was tested in jModelTest0.1 [16]. The best-fit model of nucleotide substitution was determined to be HKY+I+G [Hasegawa-Kishino-Yano model (HKY); proportion of invariable sites (I); gamma distribution shape parameter (G)] for CV-A2 strains worldwide, as well as for genotype D strains. Initially, the following three molecular clock models were tested on each dataset: the strict, uncorrelated exponential relaxed, and uncorrelated lognormal relaxed clock models. Results of the Bayes factor test indicated that the uncorrelated lognormal relaxed clock was the best fit [17]. Therefore, Bayesian Markov chain Monte Carlo methods were used to estimate the evolutionary characteristics of CV-A2 using BEAST v1.8 (http://beast.bio.ed.ac.uk/); only results based on this model are shown. Fifty to seventy million generations were run, and log and tree files were saved to generate 10,000 trees to ensure sufficient sample size (n = 1,000) for each parameter of interest. Sampling efficiency was calculated using the effective sampling size (ESS) function in TRACER v1.5 (http://tree.bio.ed.ac.uk/software/tracer/). Maximum clade credibility (MCC) dated phylogenetic trees were summarized after 10% burn-in with Tree Annotator and edited in FigTree version 1.3.1 (http://tree.bio.ed.ac.uk/software/figtree) [17]. Bayesian skyline plot depicting relative viral genetic diversity through time were generated for the D genotype CV-A2 in mainland of China in TRACER v 1.5 (http://tree.bio.ed.ac.uk/software/tracer/).

## Results

### Geographic and annual distribution of CV-A2 isolates in mainland of China

**Fig 1:**
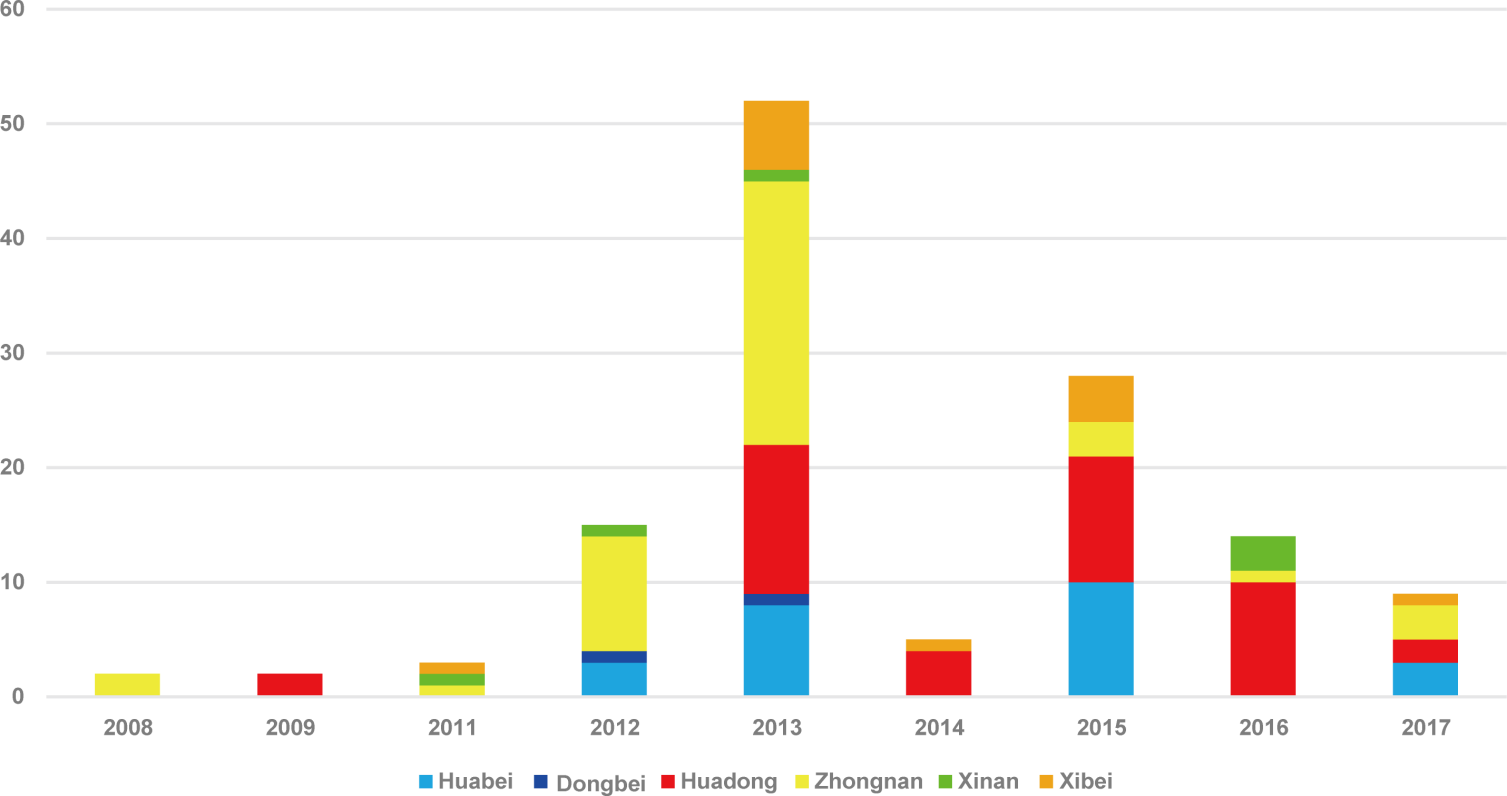
Temporal and geographical distribution of CV-A2 isolates in mainland of China from 2008 to 2017 based on the *VP1* coding region.

A total of 163 CV-A2 sequences collected in the present study and downloaded from the GenBank database were used for the analysis. Sequences information were summarized in S1 Table. A total 130 strains were isolated in mainland of China during 2008–2017 from 18 provinces, municipalities, and autonomous regions representing six administrative regions of China: East China (Shandong, Jiangsu, Zhejiang, and Jiangxi), South China (Hunan, Henan, Hainan, and Guangdong), North China (Beijing, Tianjin, Hebei), Northwest China (Ningxia, Shaanxi, Qinghai, and Gansu), Southwest China (Guizhou, and Chongqing), and Northeast China (Jilin). Only four CV-A2 isolates were obtained between 2008 and 2009; the other strains were isolated between 2011 and 2017 (Fig 1).

### Genetic characterization of the *VP1* coding region of Chinese CV-A2 isolates

Previous studies have classified CV-A2 into four genotypes, namely, genotypes A, B, C, and D, in which genotypes are distinguished based on least 15% divergence in the *VP1* nucleotide sequence [12]. Genotype C can be further divided into the C1 to C3 sub-genotypes, which exhibit more than 8% divergence in the *VP1* region [18]. Sub-genotype C1 comprises strains isolated in Australia from 2005 to 2008, in Russia from 2010 to 2011, and in the United States in 2014. Sub-genotype C2 comprises strains isolated in Russia from 2005 to 2011 and in Kazakhstan in 2010. Sub-genotype C3 comprises strains isolated in Russia, Taiwan, India, and Ukraine from 2007 to 2011. The mean nucleotide variation within each of the C sub-genotypes ranges from 6.5% (sub-genotype C3) to 8.1% (sub-genotype C2), whereas the mean nucleotide variation between different C sub-genotypes ranges from 12.8% (between sub-genotypes C1 and C2) to 14.4% (between sub-genotypes C1 and C3). Interestingly, strains isolated in Taiwan in 2008 belong to sub-genotype C3, indicating that they have different ancestors with viruses isolated in mainland of China.

**Fig 2:**
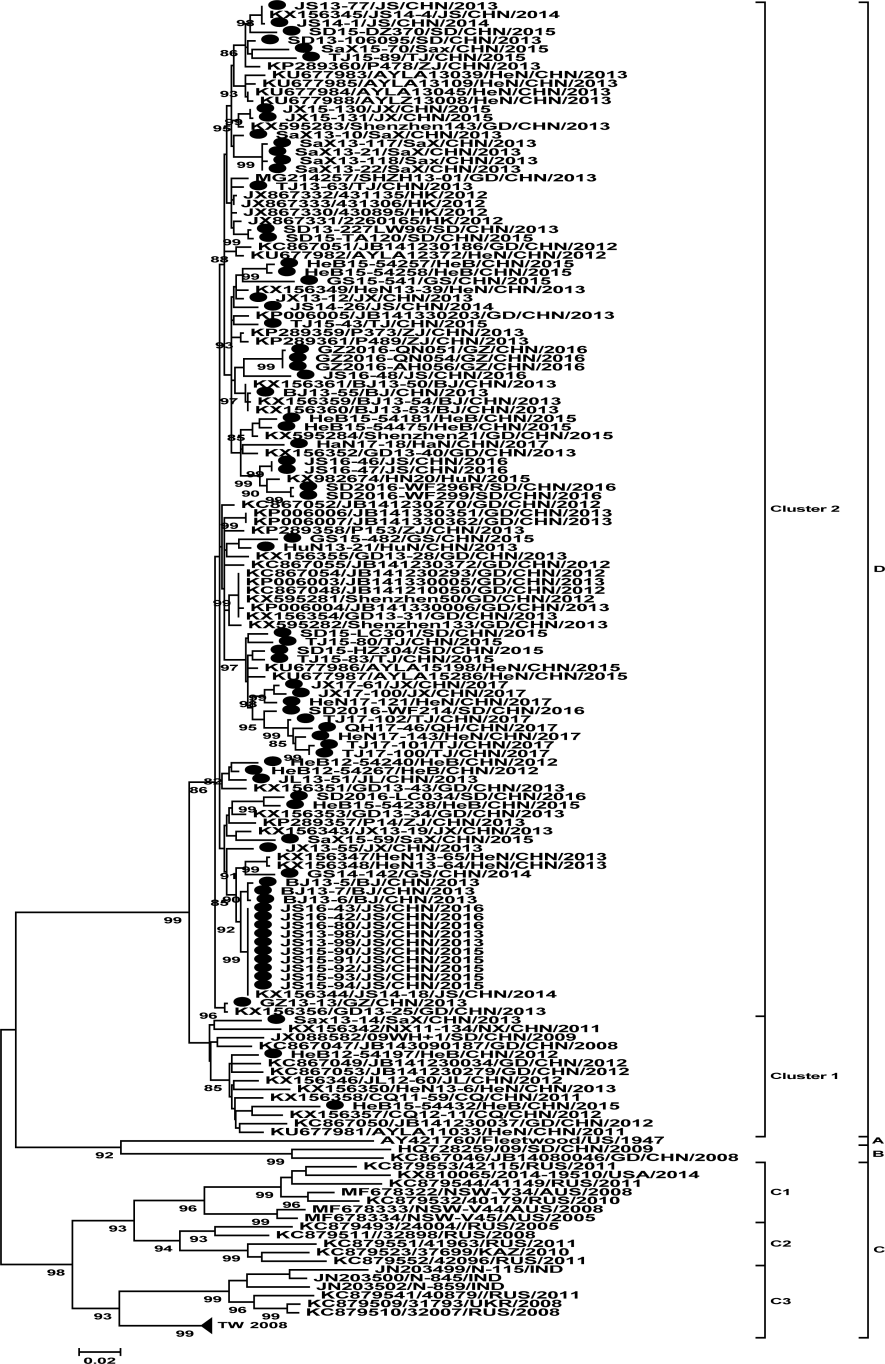
Phylogenetic trees constructed based on the entire *VP1* nucleotide sequences of CV-A2 strains isolated between 1947 and 2017. The CV-A2 strains isolated in the present study are indicated by solid circles. Numbers at the nodes indicate bootstrap support for each node (percentage of 1,000 bootstrap replicates).

As expected, all 74 strains isolated in the present study were grouped under genotype D (Fig 2), exhibiting 80.0% to 82.4% nucleotide sequence identity with the CV-A2 prototype strain (CV-A2//Fleetwood, AY421760). For genotype D, phylogenetic analysis based on entire *VP1* nucleotide sequences revealed that multiple CV-A2 lineages co-circulate in China, which could be split into two distinct clusters (clusters 1 and 2) supported by significant bootstrap values, the neighbor-joining(NJ) tree also had the same result (figure was not shown). The group mean distance between cluster 1 and cluster 2 was 6.0%. Fifteen (14/128) isolates were grouped under cluster 1 and the rest (114/128) belonged to cluster 2. Cluster 1 was composed of strains isolated in 2008–2013, except for only one strain isolated in 2015 in Hebei. The mean p-distance within cluster 1 is 4.5%. However, cluster 2 comprised strains collected between 2012 and 2017. The mean p-distance within cluster 2 is 3.2%. The data from the present study indicated that the molecular epidemiology of CV-A2 in China during the last 10 years reflected the pattern of circulation of genotype D viruses. There were two periods of genotype D CV-A2 circulation between 2008 and 2017 in mainland of China. In the first stage, from 2008 to 2012, CV-A2 belonged to cluster 1. After 2012, cluster 1 was gradually replaced by cluster 2. In the past 5 years, CV-A2 from cluster 2 became the predominant virus circulating in mainland of China. The cluster transition suggested that evolution of CV-A2 of genotype D occurred during persistent circulation in mainland of China within 10 years.

### Evolutionary analysis of CV-A2

**Fig 3:**
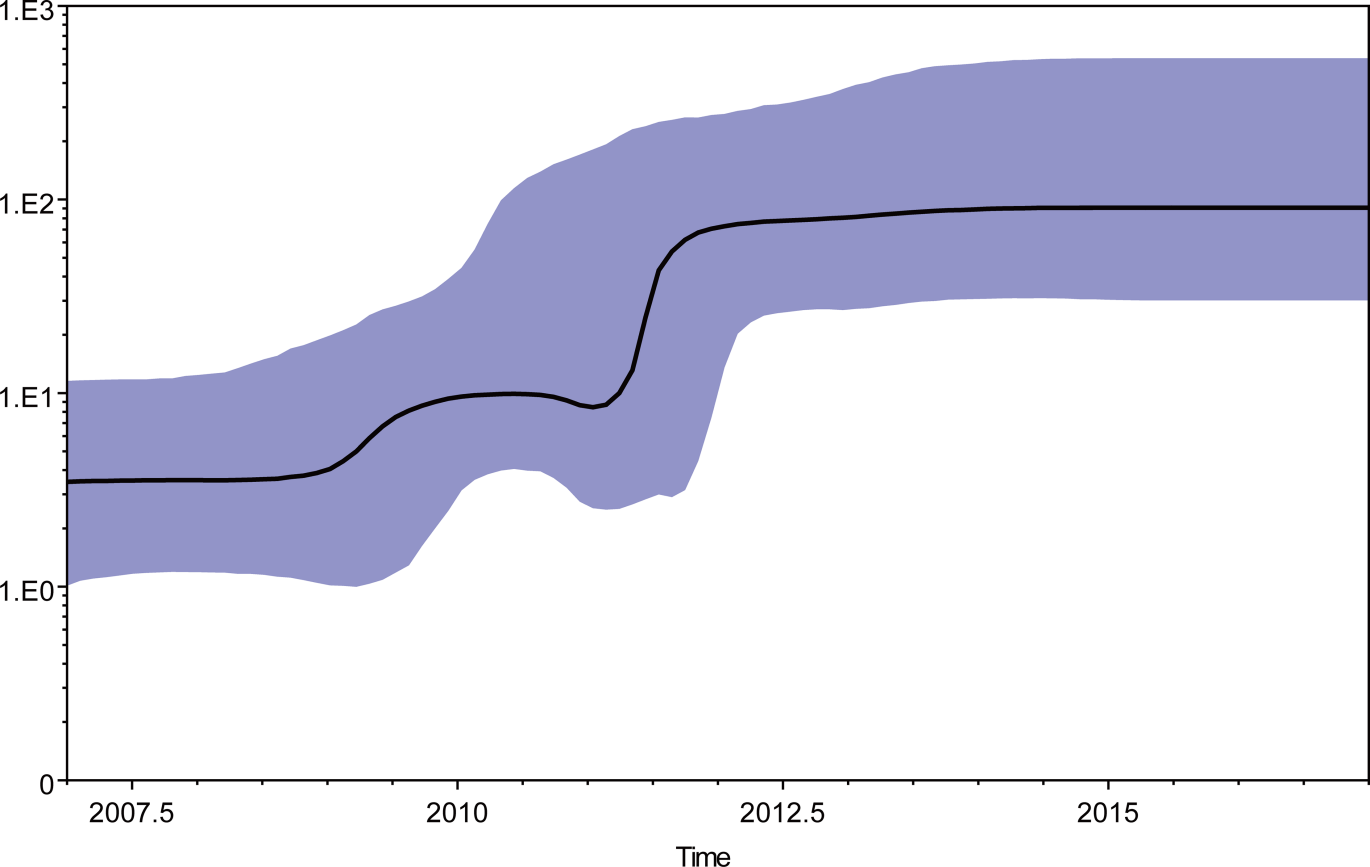
A Bayesian skyline plot analysis of the representative Chinese genotype D CV-A2 sequences from 2008–2017. Ordinate: the number of effective infections at time; abscissa: time (in years). The thick solid line represents the median, and the blue area represents the 95% highest probability density (HPD) of the number of effective infections at the time estimates.

The temporal structure of the data was tested using 163 CV-A2 sequences. Sequences information were summarized in S1 Table. A regression of root-to-tip genetic distances against sampling date was shown in S1 Fig. The correlation coefficient was 0.82 and R2 value was 0.67. It exhibited a positive correlation between genetic divergence and sampling time. The root-to-tip analysis showed that the sequences exhibited less dispersion around the best fit line compared to CV-A2 prototype strain (Fleetwood). To investigate the evolutionary history of CV-A2, MCC trees were constructed based on entire *VP1* nucleotide sequences of Chinese CV-A2 strains (n = 37) and representative strains in the ML tree (n = 16), as well as genotype D CV-A2 strains grouped (data in S1 Table). The time of the most recent common ancestor (T_MRCA_) for CV-A2 was estimated to be 1929 (95% highest probability density, 1913–1944) (data in S2 Fig). The T_MRCA_ for genotype D CV-A2 was estimated to be 2004 (95% highest probability density, 2003–2007) (data in S3 Fig). Based on our analysis, the estimated T_MRCA_ of CV-A2 was in the early 1930s, which corresponds to at least 15 years before the first reported detection of CV-A2 in 1947 [2]. The T_MRCA_ estimates suggest that genotype D viruses had been circulating in China more than 5 years before wide spread circulation in mainland of China after 2013. BEAST software was used to calculate nucleotide substitution rates for the *VP1* coding region. The mean substitution rate for the *VP1* sequence was estimated to be 5.82×10^−3^ substitutions per site per year (95% HPD, 4.52×10^−3^–7. 13×10^−3^), which is comparable to the rates previously reported for other RNA viruses. For example, the estimated substitution rates for genotype C4a of EV-A71 and CV-A6 were 4.99×10^−3^ [19] and 4.10×10^−3^ [20], respectively. The mean substitution rate for the genotype D was estimated to be 6.32×10^−3^ substitutions per site per year (95% HPD, 4.74×10^−3^–8.00×10^−3^), which is slightly higher than the reported global substitution rate for CV-A2. To estimate effective virus population sizes through time during 2008–2017, a BSP analysis was performed (Fig 3). The skyline plot showed that between 2009–2011 some genetic diversity was observed, which might be a consequence of establishment of the systematic surveillance of HFMD since 2008. Another burst of the skyline plot was observed after 2011, probably because of faster and increasing branch of evolution of CV-A2 at that time which were accordance with repeated CV-A2 outbreaks reports after 2011 [7, 8]. The genetic diversity of genotype D CV-A2 in China increased between 2011–2012. And during 2013–2017, the genetic diversity remained stable despite a decrease in number of isolates in this study.

## Discussion

CV-A2 was previously thought to cause sporadic infections with benign clinical presentation. However, deaths associated with CV-A2 have been recently reported in mainland of China. The epidemiology of CV-A2 is similar to that of EV-A71, which peaked in June and declined afterwards [5]. Another study confirmed that the peak circulation of CV-A2 occurred in July and August [21]. The prevalence of CV-A2 has been found to be associated with geographical location, seasonality, and population susceptibility. Determinants of clinical manifestation and disease severity have been linked to specific serotypes [22]. Therefore, surveillance is important for determining the etiology and transmission of viruses among young children, who are highly susceptible to infection.

The similarities between the symptoms of CV-A2 and EV-A71 infection were: both of them were the agents of HFMD, and susceptible population were younger than 3 years of age [5]. The differences were listed as follows: regarding clinical manifestation, most patients with EV-A71 infection presented with hand-foot-mouth disease, while most CV-A2 infected patients presented with herpangina. The EV-A71 infected patients had a much higher incidence of neurological signs compared with CV-A2 infected patients [5]. One study about clinical diagnosis revealed a substantial proportion of the patients in the CV-A2 infection groups noted with an elevated serum C-reactive protein level, which was higher than in the EV-A71 and CV-A16 infected patients [23].

RNA viruses are characterized by their high evolutionary rates, which is largely attributed to the low fidelity of replication in RNA viruses with error-prone RNA polymerases [24]. Estimates of evolutionary rates in RNA viruses normally fall within one order of magnitude of 1×10^−3^ nucleotide substitutions per site per year [25]. In the present study, we focused on the evolutionary analysis of CV-A2 of genotype D in mainland of China and also in the whole world. Bayesian Markov chain Monte Carlo methods were used to estimate the evolutionary characteristics of CV-A2 using BEAST software. We investigated the evolutionary dynamics of CV-A2 from all six geographic regions in mainland of China within a 10-year time span based on the disease surveillance programs for HFMD and acute flaccid paralysis (for supporting global polio eradication). To our knowledge, our study is the first to investigate the molecular evolution of CV-A2 in mainland of China. The evolutionary rate of the *VP1* sequence of genotype D CV-A2 in mainland of China was estimated to be 6.32×10^−3^ substitutions per site per year, which is comparable to the rates reported for other RNA viruses.

In this study, we expanded the surveillance scope in that collected 74 new strains which were not included in HFMD cases isolated in previous study [12], and accomplished a more comprehensive molecular epidemiological analysis of CV-A2 from all six regions in mainland of China, and analyzed based on a long time span of 2008–2017. Earlier studies have classified CV-A2 into four genotypes, genotypes A, B, C, and D. In this study, we further divided genotype C into the C1 to C3 sub-genotypes in order to better explain genotype C. According to the previous genotyping rules, all 74 strains isolated in the present study were grouped under genotype D. Interestingly, genotype D was only found in China since 2008, which need continuous surveillance.

Based on currently available data, genotype D viruses appear to be active and are widely distributed throughout China, indicating a shift from co-circulation of genotypes B and D to a predominance of genotype D. The reasons for the disappearance of genotype B viruses are unclear. Thus far, genotypes B and D have only been detected in China. Whether they have the potential to spread globally requires further investigation. Continued and improved surveillance and monitoring should be conducted to predict whether specific genotypes (or sub-genotypes) can persistently circulate for a relatively long period (similar to EV-A71 and CV-A16) [19, 26] and whether genotype D CV-A2 will continue to be the predominant causative agent of HFMD in China. Furthermore, CV-A2 is a pathogen that is critically involved in herpangina. However, herpangina is not included in the list of diseases in the compulsory reports of the disease surveillance reporting system of China. Thus, the disease burden caused by CV-A2 infections is likely to be strongly underestimated.

## Supporting information

S1 Table List of coxsackievirus A2 sequences used for analysis in this study. (DOCX)

S1 Fig Regression of root-to-tip genetic distances against sampling date. (TIF)

S2 Fig Maximum Clade Credibility (MCC) tree of complete *VP1* nucleotide sequences (885 nucleotides) of coxsackievirus A2 strains in the world. 95% HPD intervals are shown with horizontal blue bars.
(TIF)

S3 Fig Maximum Clade Credibility (MCC) tree of complete *VP1* nucleotide sequences (885 nucleotides) of genotype D coxsackievirus A2 strains in mainland of China. 95% HPD intervals are shown with horizontal blue bars.
(TIF)
