# Supplementary material for: Persistent circulation of genotype D coxsackievirus A2 in mainland of China since 2008
Source: PLoS One. 2018 Sep 20;13(9):e0204359. doi: 10.1371/journal.pone.0204359 (PMC6147602; doi:10.1371/journal.pone.0204359)
Supplement: S1 Table — (DOCX) [file pone.0204359.s001.docx]

**S1 Table. List of coxsackievirus A2 sequences used for analysis in this study**

| **Isolation year** | **Countries/region** | **Strain name** | **GenBank accession No.** | **Genotype/**  **Sub-genotype** | **Origin** | **Disease** |
| --- | --- | --- | --- | --- | --- | --- |
| 1947 | USA | Fleetwood | AY421760 | A | GenBank | AFP |
| 2008 | Guangdong,China | JB14080046 | KC867046 | B | GenBank | HFMD |
| 2009 | Shandong,China | CVA2/SD/CHN/09 | HQ728259 | B | GenBank | HFMD |
| 2008 | AUS | NSW-V34 | MF678322 | C1 | GenBank | NA |
| 2008 | AUS | NSW-V44 | MF678333 | C1 | GenBank | NA |
| 2005 | AUS | NSW-V45 | MF678334 | C1 | GenBank | NA |
| 2014 | USA | 2014-19510 | KX810065 | C1 | GenBank | NA |
| 2010 | Russia | 40179 | KC879532 | C1 | GenBank | Febrile illness |
| 2011 | Russia | 42115 | KC879553 | C1 | GenBank | Healthy |
| 2011 | Russia | 41149 | KC879544 | C1 | GenBank | GE |
| 2005 | Russia | 24004 | KC879493 | C2 | GenBank | Febrile illness |
| 2008 | Russia | 32898 | KC879511 | C2 | GenBank | AFP |
| 2010 | Russia | 37699 | KC879523 | C2 | GenBank | AFP |
| 2011 | Russia | 41963 | KC879551 | C2 | GenBank | Healthy |
| 2011 | Russia | 42096 | KC879552 | C2 | GenBank | Healthy |
| NA | India | N-859 | JN203502 | C3 | GenBank | AFP |
| NA | India | N-115 | JN203499 | C3 | GenBank | AFP |
| NA | India | N-845 | JN203500 | C3 | GenBank | AFP |
| 2008 | Russia | 31793 | KC879509 | C3 | GenBank | AFP |
| 2008 | Russia | 32007 | KC879510 | C3 | GenBank | AFP |
| 2011 | Russia | 40879 | KC879541 | C3 | GenBank | Febrile illness |
| 2008 | Taiwan,China | 61219-1463 | MF422534 | C3 | GenBank | NA |
| 2008 | Taiwan,China | 61220-1490 | MF422535 | C3 | GenBank | NA |
| 2008 | Taiwan,China | 61221-1363 | MF422536 | C3 | GenBank | NA |
| 2008 | Taiwan,China | 61222-1346 | MF422537 | C3 | GenBank | NA |
| 2008 | Taiwan,China | 61223-890 | MF422538 | C3 | GenBank | NA |
| 2008 | Taiwan,China | 61224-871 | MF422539 | C3 | GenBank | NA |
| 2008 | Taiwan,China | 61225-891 | MF422540 | C3 | GenBank | NA |
| 2008 | Taiwan,China | 61226-1413 | MF422541 | C3 | GenBank | NA |
| 2008 | Taiwan,China | 63041-775 | MF422542 | C3 | GenBank | NA |
| 2008 | Taiwan,China | 63044-244 | MF422543 | C3 | GenBank | NA |
| 2008 | Guangdong,China | JB143090187 | KC867047 | D | GenBank | HFMD |
| 2009 | Shandong,China | 09WH+1 | JX088582 | D | GenBank | AFP |
| 2011 | Ningxia,China | NX11-134/NX/CHN/2011 | KX156342 | D | GenBank | HFMD |
| 2011 | Chongqing,China | CQ11-59/CQ/CHN/2011 | KX156358 | D | GenBank | HFMD |
| 2012 | Guangdong,China | Shenzhen50 | KX595281 | D | GenBank | HA |
| 2013 | Guangdong,China | Shenzhen133 | KX595282 | D | GenBank | HA |
| 2013 | Guangdong,China | Shenzhen143 | KX595283 | D | GenBank | HA |
| 2015 | Guangdong,China | Shenzhen21 | KX595284 | D | GenBank | HFMD |
| 2013 | Guangdong,China | SHZH13-01 | MG214257 | D | GenBank | HA |
| 2013 | Jiangxi,China | JX13-19/JX/CHN/2013 | KX156343 | D | GenBank | HFMD |
| 2014 | Jiangsu,China | JS14-18/JS/CHN/2014 | KX156344 | D | GenBank | HFMD |
| 2014 | Jiangsu,China | JS14-4/JS/CHN/2014 | KX156345 | D | GenBank | HFMD |
| 2012 | Jilin,China | JL12-60/JL/CHN/2012 | KX156346 | D | GenBank | HFMD |
| 2013 | Henan,China | HeN13-65/HeN/CHN/2013 | KX156347 | D | GenBank | HFMD |
| 2013 | Henan,China | HeN13-64/HeN/CHN/2013 | KX156348 | D | GenBank | HFMD |
| 2013 | Henan,China | HeN13-39/HeN/CHN/2013 | KX156349 | D | GenBank | HFMD |
| 2013 | Henan,China | HeN13-6/HeN/CHN/2013 | KX156350 | D | GenBank | HFMD |
| 2013 | Guangdong,China | GD13-43/GD/CHN/2013 | KX156351 | D | GenBank | HFMD |
| 2013 | Guangdong,China | GD13-40/GD/CHN/2013 | KX156352 | D | GenBank | HFMD |
| 2013 | Guangdong,China | GD13-34/GD/CHN/2013 | KX156353 | D | GenBank | HFMD |
| 2013 | Guangdong,China | GD13-31/GD/CHN/2013 | KX156354 | D | GenBank | HFMD |
| 2013 | Guangdong,China | GD13-28/GD/CHN/2013 | KX156355 | D | GenBank | HFMD |
| 2013 | Guangdong,China | GD13-25/GD/CHN/2013 | KX156356 | D | GenBank | HFMD |
| 2012 | Chongqing,China | CQ12-11/CQ/CHN/2012 | KX156357 | D | GenBank | HFMD |
| 2013 | Beijing ,China | BJ13-54/BJ/CHN/2013 | KX156359 | D | GenBank | HFMD |
| 2013 | Beijing ,China | BJ13-53/BJ/CHN/2013 | KX156360 | D | GenBank | HFMD |
| 2013 | Beijing ,China | BJ13-50/BJ/CHN/2013 | KX156361 | D | GenBank | HFMD |
| 2013 | Zhejiang,China | P489 | KP289361 | D | GenBank | HFMD |
| 2013 | Zhejiang,China | P373 | KP289359 | D | GenBank | HFMD |
| 2013 | Zhejiang,China | P478 | KP289360 | D | GenBank | HFMD |
| 2013 | Zhejiang,China | P153 | KP289358 | D | GenBank | HFMD |
| 2013 | Zhejiang,China | P14 | KP289357 | D | GenBank | HFMD |
| 2012 | Hongkong,China | 2260165 | JX867331 | D | GenBank | RTI |
| 2012 | Hongkong,China | 430895 | JX867330 | D | GenBank | RTI |
| 2012 | Hongkong,China | 431135 | JX867332 | D | GenBank | RTI |
| 2012 | Hongkong,China | 431306 | JX867333 | D | GenBank | RTI |
| 2012 | Guangdong,China | JB141230186 | KC867051 | D | GenBank | HFMD |
| 2012 | Guangdong,China | JB141230270 | KC867052 | D | GenBank | HFMD |
| 2012 | Guangdong,China | JB141230372 | KC867055 | D | GenBank | HFMD |
| 2013 | Guangdong,China | JB141330005 | KP006003 | D | GenBank | NA |
| 2013 | Guangdong,China | JB141330006 | KP006004 | D | GenBank | NA |
| 2013 | Guangdong,China | JB141330203 | KP006005 | D | GenBank | NA |
| 2013 | Guangdong,China | JB141330351 | KP006006 | D | GenBank | NA |
| 2013 | Guangdong,China | JB141330362 | KP006007 | D | GenBank | NA |
| 2012 | Guangdong,China | JB141210050 | KC867048 | D | GenBank | HFMD |
| 2012 | Guangdong,China | JB141230293 | KC867054 | D | GenBank | HFMD |
| 2016 | Hunan,China | HN20 | KX982674 | D | GenBank | NA |
| 2012 | Guangdong,China | JB141230037 | KC867050 | D | GenBank | HFMD |
| 2012 | Guangdong,China | JB141230034 | KC867049 | D | GenBank | HFMD |
| 2012 | Guangdong,China | JB141230279 | KC867053 | D | GenBank | HFMD |
| 2013 | Henan,China | AYLZ13008 | KU677988 | D | GenBank | NA |
| 2015 | Henan,China | AYLA15286 | KU677987 | D | GenBank | NA |
| 2015 | Henan,China | AYLA15198 | KU677986 | D | GenBank | NA |
| 2013 | Henan,China | AYLA13109 | KU677985 | D | GenBank | NA |
| 2013 | Henan,China | AYLA13045 | KU677984 | D | GenBank | NA |
| 2013 | Henan,China | AYLA13039 | KU677983 | D | GenBank | NA |
| 2012 | Henan,China | AYLA12372 | KU677982 | D | GenBank | NA |
| 2011 | Henan,China | AYLA11033 | KU677981 | D | GenBank | NA |
| 2012 | Hebei,China | HeB12-54197/HeB/CHN/2012 |  | D | This study | HFMD |
| 2012 | Hebei,China | HeB12-54240/HeB/CHN/2012 |  | D | This study | HFMD |
| 2012 | Hebei,China | HeB12-54267/HeB/CHN/2012 |  | D | This study | HFMD |
| 2013 | Beijing ,China | BJ13-5/BJ/CHN/2013 |  | D | This study | HFMD |
| 2013 | Beijing ,China | BJ13-6/BJ/CHN/2013 |  | D | This study | HFMD |
| 2013 | Beijing ,China | BJ13-7/BJ/CHN/2013 |  | D | This study | HFMD |
| 2013 | Beijing ,China | BJ13-55/BJ/CHN/2013 |  | D | This study | HFMD |
| 2013 | Guizhou,China | GZ13-13/GZ/CHN/2013 |  | D | This study | HFMD |
| 2013 | Hunan,China | HuN13-21/HuN/CHN/2013 |  | D | This study | HFMD |
| 2013 | Jilin,China | JL13-51/JL/CHN/2013 |  | D | This study | HFMD |
| 2013 | Jiangsu,China | JS13-77/JS/CHN/2013 |  | D | This study | HFMD |
| 2013 | Jiangsu,China | JS13-98/ JS/CHN/2013 |  | D | This study | HFMD |
| 2013 | Jiangsu,China | JS13-99/ JS/CHN/2013 |  | D | This study | HFMD |
| 2013 | Jiangxi,China | JX13-12/JX/CHN/2013 |  | D | This study | HFMD |
| 2013 | Jiangxi,China | JX13-55/JX/CHN/2013 |  | D | This study | HFMD |
| 2013 | Shaanxi,China | SaX13-10/SaX/CHN/2013 |  | D | This study | HFMD |
| 2013 | Shaanxi,China | SaX13-14/SaX/CHN/2013 |  | D | This study | HFMD |
| 2013 | Shaanxi,China | SaX13-21/SaX/CHN/2013 |  | D | This study | HFMD |
| 2013 | Shaanxi,China | SaX13-22/SaX/CHN/2013 |  | D | This study | HFMD |
| 2013 | Shaanxi,China | SaX13-117/SaX/CHN/2013 |  | D | This study | HFMD |
| 2013 | Shaanxi,China | SaX13-118/SaX/CHN/2013 |  | D | This study | HFMD |
| 2013 | Shandong,China | SD13-227LW96/SD/CHN/2013 |  | D | This study | HFMD |
| 2013 | Shandong,China | SD13-106095/SD/CHN/2013 |  | D | This study | HFMD |
| 2013 | Tianjin,China | TJ13-63/TJ/CHN/2013 |  | D | This study | HFMD |
| 2014 | Gansu, China | GS14-142/GS/CHN/2014 |  | D | This study | HFMD |
| 2014 | Jiangsu,China | JS14-1/JS/CHN/2014 |  | D | This study | HFMD |
| 2014 | Jiangsu,China | JS14-26/JS/CHN/2014 |  | D | This study | HFMD |
| 2015 | Gansu, China | GS15-482/GS/CHN/2015 |  | D | This study | HFMD |
| 2015 | Hebei,China | HeB15-54181/HeB/CHN/2015 |  | D | This study | HFMD |
| 2015 | Hebei,China | HeB15-54238/ HeB/CHN/2015 |  | D | This study | HFMD |
| 2015 | Hebei,China | HeB15-54257/ HeB/CHN/2015 |  | D | This study | HFMD |
| 2015 | Hebei,China | HeB15-54258/ HeB/CHN/2015 |  | D | This study | HFMD |
| 2015 | Hebei,China | HeB15-54432/ HeB/CHN/2015 |  | D | This study | HFMD |
| 2015 | Hebei,China | HeB15-54475/ HeB/CHN/2015 |  | D | This study | HFMD |
| 2015 | Jiangxi,China | JX15-130/JX/CHN/2015 |  | D | This study | HFMD |
| 2015 | Jiangxi,China | JX15-131/JX/CHN/2015 |  | D | This study | HFMD |
| 2015 | Shaanxi,China | SaX15-59/SaX/CHN/2015 |  | D | This study | HFMD |
| 2015 | Shaanxi,China | SaX15-70/ SaX/CHN/2015 |  | D | This study | HFMD |
| 2015 | Shandong,China | SD15-DZ370/SD/CHN/2015 |  | D | This study | HFMD |
| 2015 | Shandong,China | SD15-HZ304/SD/CHN/2015 |  | D | This study | HFMD |
| 2015 | Shandong,China | SD15-LC301/SD/CHN/2015 |  | D | This study | HFMD |
| 2015 | Shandong,China | SD15-TA120/SD/CHN/2015 |  | D | This study | HFMD |
| 2015 | Tianjin,China | TJ15-43/TJ/CHN/2015 |  | D | This study | HFMD |
| 2015 | Tianjin,China | TJ15-80/TJ/CHN/2015 |  | D | This study | HFMD |
| 2015 | Tianjin,China | TJ15-83/TJ/CHN/2015 |  | D | This study | HFMD |
| 2015 | Tianjin,China | TJ15-89/TJ/CHN/2015 |  | D | This study | HFMD |
| 2015 | Jiangsu,China | JS15-90/JS/CHN/2015 |  | D | This study | HFMD |
| 2015 | Jiangsu,China | JS15-91/JS/CHN/2015 |  | D | This study | HFMD |
| 2015 | Jiangsu,China | JS15-92/JS/CHN/2015 |  | D | This study | HFMD |
| 2015 | Jiangsu,China | JS15-93/JS/CHN/2015 |  | D | This study | HFMD |
| 2015 | Jiangsu,China | JS15-94/JS/CHN/2015 |  | D | This study | HFMD |
| 2015 | Gansu, China | GS15-541/GS/CHN/2015 |  | D | This study | HFMD |
| 2016 | Guizhou,China | GZ2016-AH056/GZ/CHN/2016 |  | D | This study | HFMD |
| 2016 | Guizhou,China | GZ2016-QN051/GZ/CHN/2016 |  | D | This study | HFMD |
| 2016 | Guizhou,China | GZ2016-QN054/GZ/CHN/2016 |  | D | This study | HFMD |
| 2016 | Shandong,China | SD2016-LC034/SD/CHN/2016 |  | D | This study | HFMD |
| 2016 | Shandong,China | SD2016-WF214/SD/CHN/2016 |  | D | This study | HFMD |
| 2016 | Shandong,China | SD2016-WF296R/SD/CHN/2016 |  | D | This study | HFMD |
| 2016 | Shandong,China | SD2016-WF299/SD/CHN/2016 |  | D | This study | HFMD |
| 2016 | Jiangsu,China | JS16-42/JS/CHN/2016 |  | D | This study | HFMD |
| 2016 | Jiangsu,China | JS16-43/JS/CHN/2016 |  | D | This study | HFMD |
| 2016 | Jiangsu,China | JS16-46/JS/CHN/2016 |  | D | This study | HFMD |
| 2016 | Jiangsu,China | JS16-47/JS/CHN/2016 |  | D | This study | HFMD |
| 2016 | Jiangsu,China | JS16-48/JS/CHN/2016 |  | D | This study | HFMD |
| 2016 | Jiangsu,China | JS16-80/JS/CHN/2016 |  | D | This study | HFMD |
| 2017 | Hainan,China | HaN17-18/HaN/CHN/2017 |  | D | This study | HFMD |
| 2017 | Jiangxi,China | JX17-61/JX/CHN/2017 |  | D | This study | HFMD |
| 2017 | Jiangxi,China | JX17-100/JX/CHN/2017 |  | D | This study | HFMD |
| 2017 | Qinghai,China | QH17-46/QH/CHN/2017 |  | D | This study | HFMD |
| 2017 | Tianjin,China | TJ17-100/TJ/CHN/2017 |  | D | This study | HFMD |
| 2017 | Tianjin,China | TJ17-101/ TJ/CHN/2017 |  | D | This study | HFMD |
| 2017 | Tianjin,China | TJ17-102 TJ//CHN/2017 |  | D | This study | HFMD |
| 2017 | Henan,China | HeN17-121/HeN/CHN/2017 |  | D | This study | HFMD |
| 2017 | Henan,China | HeN17-143/HeN/CHN/2017 |  | D | This study | HFMD |

AFP – acute flaccid paralysis.

All not-specific general and respiratory diagnoses were grouped under “febrile illness”. GE -gastroenteritis

HA- herpangina

HFMD- hand, foot, and mouth disease

RTI- respiratory tract infection

The red labelled sequences were used to for evolutionary analysis.
