# Supplementary figures and images for: Persistent circulation of genotype D coxsackievirus A2 in mainland of China since 2008
Source: PLoS One. 2018 Sep 20;13(9):e0204359. doi: 10.1371/journal.pone.0204359 (PMC6147602; doi:10.1371/journal.pone.0204359)

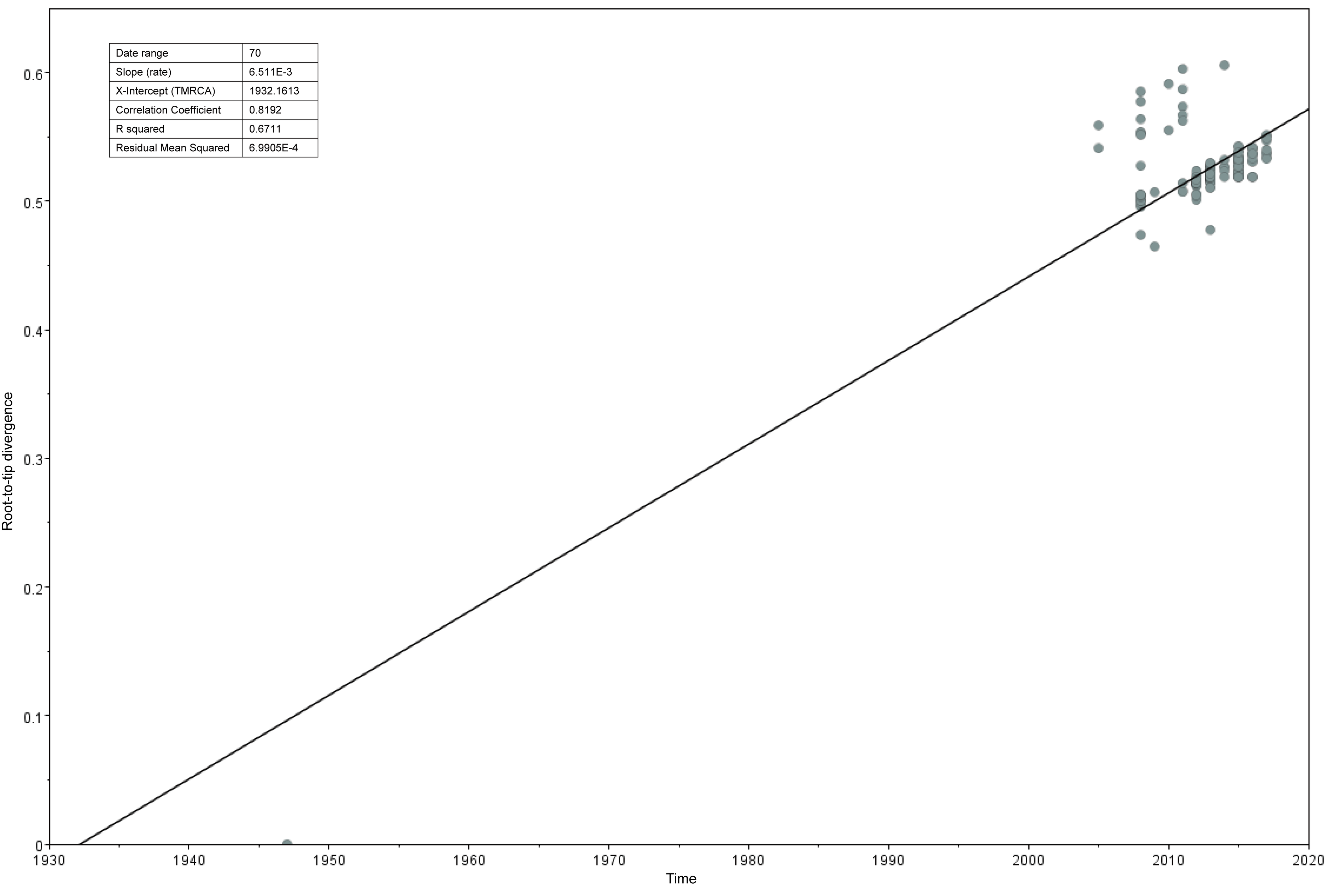

Supplement: S1 Fig — (TIF) [file pone.0204359.s002.tif]

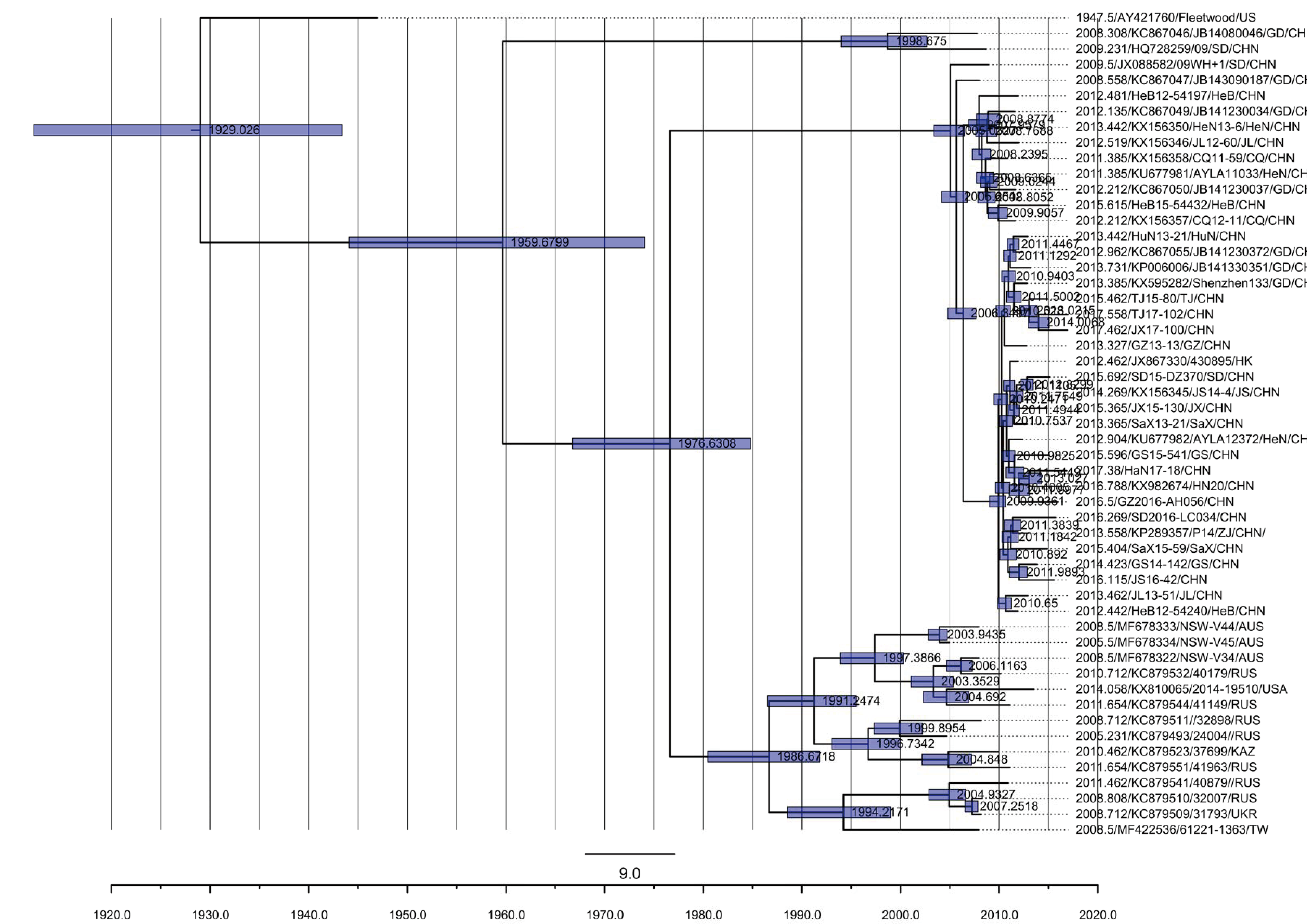

Supplement: S2 Fig — 95% HPD intervals are shown with horizontal blue bars. (TIF) [file pone.0204359.s003.tif]

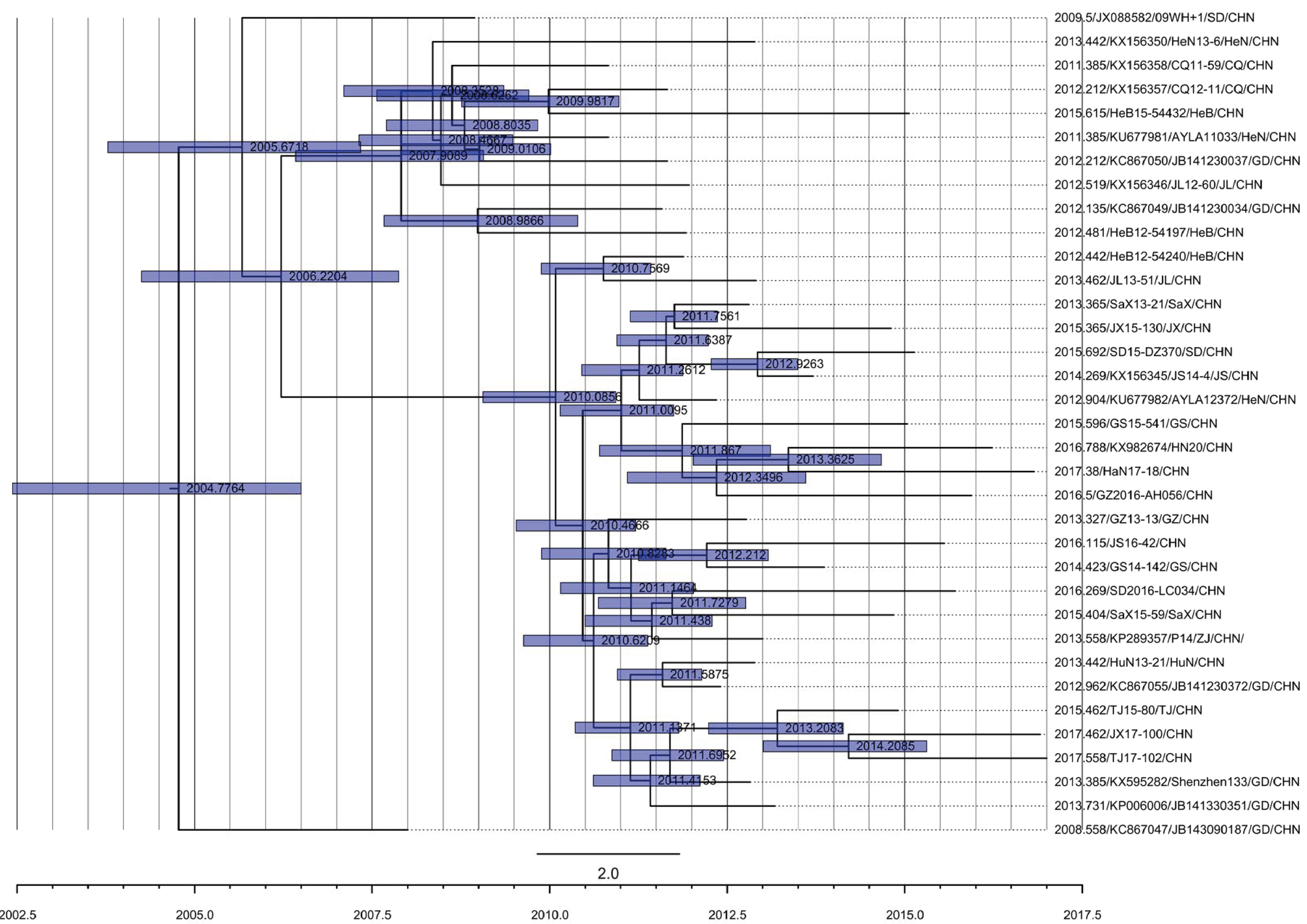

Supplement: S3 Fig — 95% HPD intervals are shown with horizontal blue bars. (TIF) [file pone.0204359.s004.tif]
